# Inter-rater agreement of the triagesystem RETTS-HEV

**DOI:** 10.1186/1757-7241-21-S2-A32

**Published:** 2013-09-09

**Authors:** Louise Nissen, Hans Kirkegaard, Noel Perez, Ulf Hørlyck, Louise Pape

**Affiliations:** 1 Emergency Department, Regionshospital Herning hospital, Denmark; 2 Research Center for Emergency Medicine, Aarhus University Hospital, Denmark; 3 Dept. of Occupational Medicine,Regional Hospital Herning, Denmark

## Background

The purpose of this study was to evaluate the inter-rater agreement among nurses using the triage system REETS- HEV (rapid emergency triage and treatment system-hospitalsenheden vest) in a Danish ED.

The use of triage systems in Denmark has recently been implemented together with structural changes in hospital organization .Testing and evaluation is therefore needed. The REETS-HEV is a five scale triage system being used in the Emergency department (ED) of Herning, Denmark since May 2010. The ED is semi-large with 29,000 annual visits.

## Methods

Consecutive patients presenting to the ED were assessed by both a duty and study nurse using REETS-HEV. Nurses did not receive training prior to the study. 146 patients were enrolled and a blinded, paired and simultaneous triage was conducted independently to evaluate inter-rater agreement using Fleiss kappa.

## Results

A total of 155 patients were triaged over a 10 day period and complete data were available for 146 patients. We found the overall agreement to be good (Fleiss kappa 0.60 (0.48; 0.72)). The kappa estimate was higher for the group of patients needing immediate attention (0.83 (0.18;1.47)).

## Conclusion

The study demonstrated good inter-rater agreement between two independent observers not receiving any new triage training prior to the study.

